# Noninvasive diffuse optical monitoring of cerebral blood flow and oxygenation responses to intermittent hypoxia in neonatal rats

**DOI:** 10.1117/1.JBO.31.4.047001

**Published:** 2026-04-16

**Authors:** Pegah Safavi, Mehrana Mohtasebi, Chowdhury Azimul Haque, Faezeh Akbari, Xuhui Liu, Yiqi Yuan, Li Chen, Lei Chen, Guoqiang Yu

**Affiliations:** a University of Kentucky, Department of Biomedical Engineering, Lexington, Kentucky, United States; b Bioptics Technology LLC, Advanced Science & Technology Commercialization Center (ASTeCC), Lexington, Kentucky, United States; c University of Kentucky, Department of Internal Medicine, Lexington, Kentucky, United States; d University of Kentucky, Department of Neurosurgery, Lexington, Kentucky, United States

**Keywords:** diffuse laser speckle contrast, cerebral blood flow, cerebral blood oxygenation, preterm infant, intermittent hypoxia, brain injury

## Abstract

**Significance:**

Intermittent hypoxia (IH) is common in preterm neonates and can cause hypoxic–ischemic brain injury. Simultaneous monitoring of cerebral blood flow (CBF) and oxygenation is essential to detect oxygen delivery-extraction mismatches and guide intervention.

**Aim:**

We aimed to adapt and test an innovative diffuse speckle contrast flow oximetry (DSCFO) system for continuous monitoring of cerebral hemodynamics during IH in neonatal rats, a model approximating human neonates.

**Approach:**

Two compact laser diodes and a miniature CMOS camera were integrated into a fiber-free probe for continuous monitoring of changes in relative CBF (rCBF) and oxy- and deoxy-hemoglobin concentrations (Δ[HbO_2_] and Δ[Hb]) in 8-day-old neonatal rats. Sham rats (n=6) underwent 10 min of normoxia, whereas IH rats (n=8) experienced 10 cycles of sequential 2-min hypoxia (8% O_2_) and 2-min hyperoxia (100% $O_{2}$).

**Results:**

Sham rats maintained stable cerebral hemodynamics under normoxia, whereas IH rats exhibited pronounced periodic fluctuations during IH. Hypoxic episodes caused instantaneous decreases in rCBF and $\Delta [{\mathrm{HbO}}_{2}]$ and increases in Δ[Hb], whereas hyperoxic episodes reversed these effects, reducing hypoxic stress. However, the pronounced cerebral hemodynamic fluctuations during IH may still contribute to brain injury.

**Conclusions:**

We demonstrate an affordable, noninvasive, wearable, fiber-free DSCFO system for continuous monitoring of rCBF, $\Delta [{\mathrm{HbO}}_{2}]$, and Δ[Hb] during IH in neonatal rats. The system captured hypoxia-induced cerebral deoxygenation and hyperoxia-driven recovery, revealing episode-dependent protective and disruptive mechanisms. Future work will correlate DSCFO findings with neurological and histological outcomes to guide IH interventions in large neonatal animal models and human infants.

## 1 Introduction

Preterm birth remains a critical global health concern, with infants delivered before reaching 37 weeks of gestation facing significantly increased risks of both morbidity and mortality. This vulnerability is even more pronounced for those born extremely preterm, defined as birth before 28 weeks of gestation.^1^^–^^3^ One of the major challenges associated with premature birth is the underdevelopment of the respiratory system, which often results in instability in breathing patterns. This immaturity contributes to conditions such as apnea of prematurity, affecting over 85% of extremely preterm infants, as well as respiratory complications in nearly 30% of preterm neonates.^4^^,^^5^ As a result, these respiratory complications frequently lead to recurring episodes of intermittent hypoxia (IH), a condition that impacts ∼94% of extremely preterm infants.^6^

IH is characterized by transient declines in arterial blood oxygen saturation (${\mathrm{SpO}}_{2}$), which is commonly monitored using peripheral pulse oximetry.^7^ Given the high prevalence of IH in preterm infants, especially those born extremely preterm, it is critical to consider its effects beyond respiratory function. The brain is highly sensitive to changes in oxygen levels, and fluctuations in cerebral blood flow (CBF) and cerebral oxygenation during IH episodes can have lasting consequences on neonatal brain development.^8^^,^^9^ Continuous monitoring of IH and its impact on cerebral hemodynamics is crucial for understanding its effects on neurodevelopment and informing potential interventions.

A variety of portable devices have been developed for noninvasive and continuous cerebral monitoring of neonates at the bedside in clinical settings. Transcranial Doppler ultrasound provides real-time CBF velocity measurements in large vessels but is limited by its large probe size and sensitivity to movement, restricting its use for long-term CBF monitoring in the brain microvasculature. Near-infrared spectroscopy (NIRS) has been used for continuous monitoring of cerebral oxygenation in preterm infants with IH.^10^^–^^14^ Conventional NIRS measures light attenuations by tissue absorption (primarily hemoglobin in red blood cells) and scattering at multiple wavelengths to calculate changes in oxy- and deoxy-hemoglobin concentrations ([${\mathrm{HbO}}_{2}$ and [Hb]).^10^^,^^11^^,^^15^^–^^17^ More recently, wearable NIRS systems enable continuous monitoring of cerebral oxygenation in freely behaving subjects.^18^^–^^21^ However, the clinical utility of NIRS is often inconclusive because cerebral oxygenation level reflects hemodynamic changes in both oxygen supply (via CBF) and oxygen consumption. To disentangle the different contributions, both CBF and cerebral oxygenation must be measured.^22^^–^^24^

Another dynamic NIRS method, diffuse correlation spectroscopy (DCS), enables noninvasive CBF measurements.^25^^–^^31^ DCS employs coherent near-infrared light and single-photon counting avalanche photodiodes (APDs) to detect temporal fluctuations of diffuse laser speckles caused by red blood cell movements in the brain (i.e., CBF). Hybrid NIRS/DCS instruments have been developed to simultaneously measure CBF and cerebral oxygenation.^22^^–^^24^^,^^27^^,^^29^^,^^32^^–^^37^ Although effective, DCS relies on large, expensive, discrete long-coherence lasers and APDs coupled to fiber-optic probes, which are heavy, fragile (posing safety concerns), susceptible to motion artifacts, and restrictive to subject movement.

To address the limitations of existing techniques, we have developed a low-cost, wearable, and fiber-free diffuse speckle contrast flowmetry (DSCF) technology for continuous and longitudinal monitoring of CBF variations in anesthetized and freely behaving subjects.^38^^–^^41^ Unlike DCS, DSCF uses a low-cost laser diode as a point source and a compact complementary metal oxide semiconductor (CMOS) camera as a two-dimensional (2D) detector to detect spatial fluctuations of diffuse laser speckles, thus improving the sampling rate, increasing the signal-to-noise ratio (SNR) through spatial averaging, and reducing overall system costs. More recently, we have extended the single-wavelength DSCF to a dual-wavelength diffuse speckle contrast flow oximetry (DSCFO, U.S. Patent No. 10/842,422, 2020) system for simultaneous monitoring of CBF and cerebral oxygenation variations.^42^ Like DSCF, DSCFO incorporates a wearable, fiber-free probe with small laser diodes and a compact CMOS camera, connected to a portable device via flexible electrical wiring. Our DSCFO system has been evaluated in tissue-simulating phantoms, human forearms, and brains of neonatal piglets and human preterm infants under diverse pathophysiological conditions.^43^^,^^44^

Given the high prevalence of IH in preterm infants and its potential impact on neurodevelopment, the present study aims to establish the DSCFO as a reliable tool for tracking cerebral hemodynamic responses in a neonatal rat model of IH. Neonatal rats were selected in this study for their cost-effectiveness and ease of handling. To achieve this goal, the DSCFO probe and device were miniaturized and optimized for use in small neonatal rats during IH. The optimized DSCFO system successfully captured dynamic fluctuations in CBF, [${\mathrm{HbO}}_{2}$], and [Hb] during IH, demonstrating its sensitivity to hypoxia-induced cerebral hemodynamic alterations. Notably, this is the first study to continuously and noninvasively monitor IH-induced changes in both CBF and cerebral oxygenation in neonatal rodents, providing valuable insights into the impact of IH on neonatal cerebral hemodynamics. This advancement provides a scalable DSCFO solution for neonatal brain research. It also lays the foundation for future clinical translation, particularly in neonatal intensive care units (NICUs), where continuous cerebral monitoring may help detect and manage hypoxia-induced brain injury in preterm infants.

## 2 Methods

### 2.1 Optimized DSCFO System for Neonatal Rats

**Fig. 1:**
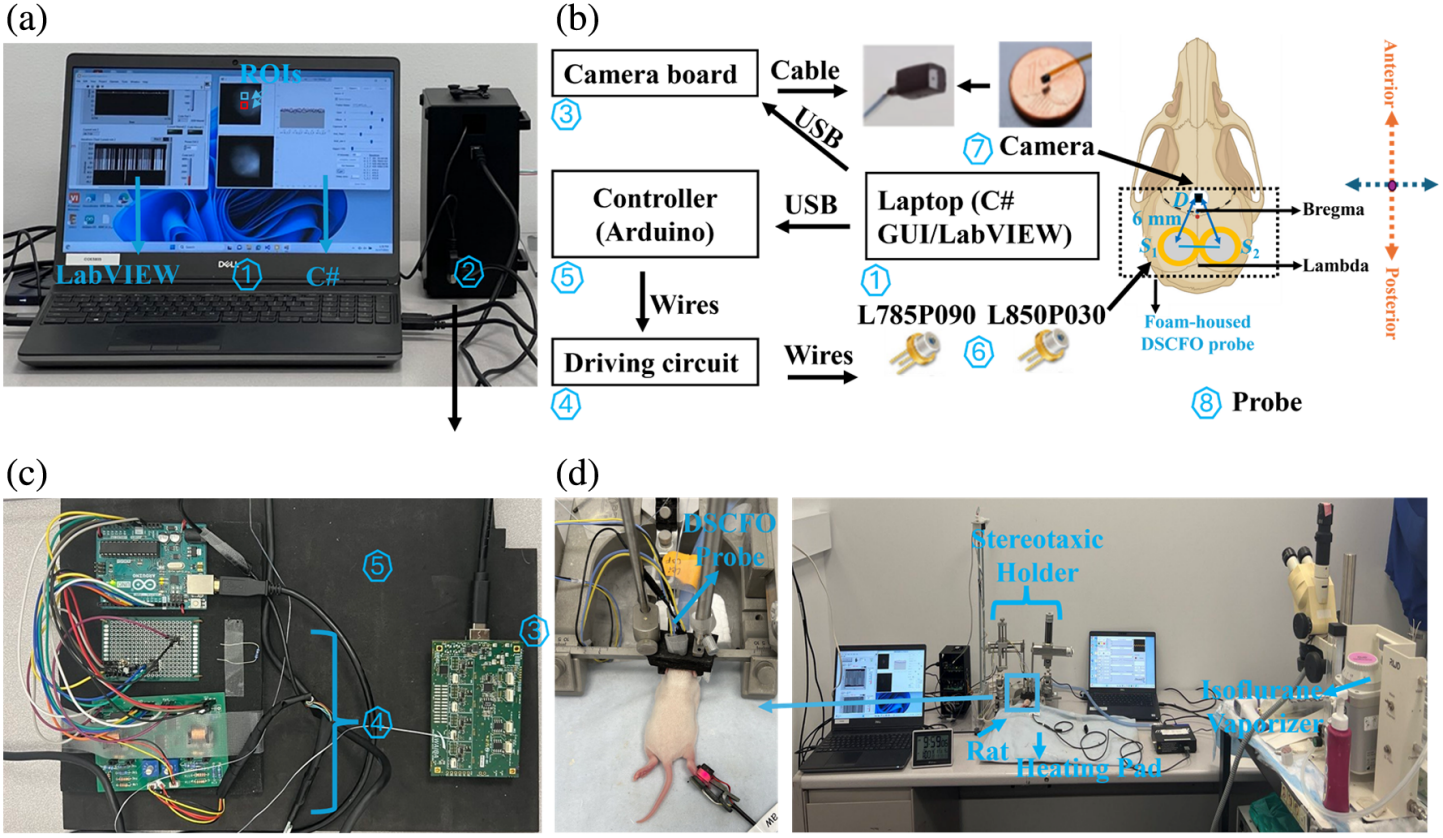
Dual-wavelength DSCFO system for simultaneous monitoring of CBF and cerebral oxygenation. (a) User interface: (1) LabVIEW^TM^ and C# GUIs and (2) DSCFO device. (b) DSCFO system diagram, including the (3) commercial camera interface board, (4) laser diode driving circuit, (5) Arduino controller, (6) laser diodes ($S_{1}=785\;\mathrm{nm}$ and $S_{2}=850\;\mathrm{nm}$), and (7) NanEye2D camera. The laser diodes and camera were configured with a 6-mm S-D separation in the (8) probe. (c) Components of the DSCFO device. (d) DSCFO probe installed on the neonatal rat head (left) and experimental setup (right).

Details for DSCFO technology can be found in our previous publications.^44^^,^^45^ Figure 1 shows the optimized DSCFO system, including the hardware and software for simultaneous monitoring of CBF and cerebral oxygenation variations in neonatal rats during IH.

#### 2.1.1 DSCFO hardware

Figure 1(a) shows the DSCFO system, which includes a graphical user interface (GUI) displayed on a laptop (1) and the DSCFO device (2). Figures 1(b) and 1(c) show the diagram and components of the DSCFO device, including a commercial camera interface board (3), a customized dual-channel laser diode driving circuit (4), and a microcontroller (Arduino Uno, SparkFun, Niwot, Colorado, United States) (5). The entire device assembly was enclosed in an electrically shielded housing and communicated with the laptop via two USB cables for power supply and data transfer. Specifically, a customized dual-channel circuit board was designed to drive two small laser diodes at 785 nm and 850 nm (6; L785P090, Ø5.6  mm, 90 mW; L850P030, Ø5.6  mm, 30 mW, Thorlabs, Newton, New Jersey, United States). A built-in photodiode in each laser diode package continuously monitored the emitted light intensity, and accordingly, the customized feedback circuit adjusted the driving current to ensure output stability. The driver board was directly connected to the Arduino Uno to enable automatically programmable control.

An ultra-small, low-power NanEye2D RGB camera (7; dimensions: $1\times 1\;{\mathrm{mm}}^{2}$; resolution: 249×250  pixels; pixel size: $3\times 3\;{\mathrm{\mu m}}^{2}$; fastest frame rate: 50 Hz; power consumption: 4 mW, Awaiba, Yverdon-les-Bains, Switzerland) was used as a 2D detector array for detecting diffuse speckle contrasts. The camera included an integrated lens (FOV 90 degrees, *F*/# 2.7) with a 0.66-mm effective focal length, providing a $4\times 4\;{\mathrm{mm}}^{2}$ field of view at a ∼5  mm working distance, and was operated through the camera board.

Figure 1(d) shows DSCFO measurement in a neonatal rat using a fiber-free probe (8; dimensions: 33  mm×20  mm×0.5  mm) mounted on the rat head and secured with stereotaxic holders. The probe included two laser diodes (S1 = 785 nm and S2 = 850 nm) and a NanEye2D camera (D), arranged with the source-detector (S-D) separation of 6 mm to achieve a penetration depth of ∼3  mm (half of the S-D separation),^26^^,^^46^^–^^48^ sufficient to probe the neonatal rat brain. The NanEye2D camera was enclosed in a transparent protective film and inserted into a clear plastic tube, whereas the laser diodes were left exposed to allow air circulation for heat dissipation.

#### 2.1.2 DSCFO software

A GUI was developed in Microsoft C# to facilitate real-time control of the camera and image visualization [Fig. 1(a)]. The C# program allowed users to configure camera parameters and select regions of interest (ROIs). In addition, a LabVIEW™ (National Instruments, Austin, Texas, United States) program was implemented to preset, monitor, and automatically stabilize laser diode output intensities. The LabVIEW™ program employed a LINX module and application logic to mediate communication between the laptop and Arduino Uno. Synchronization between the camera and the laser control/stabilization module was achieved via Transmission Control Protocol/Internet Protocol communication between the C# and LabVIEW^TM^ programs, using the laptop’s loopback address.

#### 2.1.3 DSCFO optimization

Several strategies were implemented to optimize the system configuration and performance for this application. Ideally, the dual-wavelength DSCFO system can achieve a sampling rate of up to 25 Hz when operating at the camera’s maximum frame rate of 50 Hz. However, practical constraints such as laser diode switching time and the need for intensity stabilization and synchronization limit the achievable sampling rate. This study selected a sampling rate of ∼1.6  Hz to balance between the temporal resolution and SNR. This sampling rate was sufficient for continuous monitoring of CBF and cerebral oxygenation dynamics during recurrent IH episodes.

For dual-wavelength intensity and speckle contrast analyses of blood flow and oxygenation, only red-filtered pixels from the Bayer-patterned NanEye2D RGB sensor were used, as they showed the highest sensitivity at the operational near-infrared wavelengths (785 and 850 nm). Although the NanEye2D sensor captures native 10-bit data, its driver interpolates the output to a 16-bit format. Although this does not increase the true dynamic range, it enhances numerical precision by enabling finer gradation of pixel intensity values, which benefits speckle contrast analysis.

Based on preliminary calibration tests, the NanEye2D camera was configured with an internal amplification gain setting of 2, which balanced between signal amplification and noise minimization. To further enhance signal stability, the analog-to-digital converter (ADC) offset was set to 1.6 V (offset = 3) to compensate for sensor drift. This configuration was determined after systematically evaluating all combinations of gain (0 to 3, corresponding to −1.6 to 6.5 dB) and offset (0 to 3) settings. The optimal performance, with the highest SNR, was achieved using gain = 2 and offset = 3.^45^ All measurements used a camera exposure time of 5 ms.

Accurate CBF quantification via speckle contrast requires correcting for dark current, reading noise, shot noise, and quantization noise. As speckle contrast is computed in digital numbers (DNs), the camera conversion gain (*g*, $\mathrm{DN}/e^{-}$) is used to convert DN to electrons for proper noise estimation and correction. The theoretical conversion gain under our setting was $4.85\;\mathrm{DN}/e^{-}$ calculated as *g* = ADC/full-well capacity (FWC). Here, ADC corresponds to 65,535 DN for a 16-bit depth, and FWC is $13,500\;e^{-}$ according to the camera data sheet.^45^^,^^46^

**Fig. 2:**
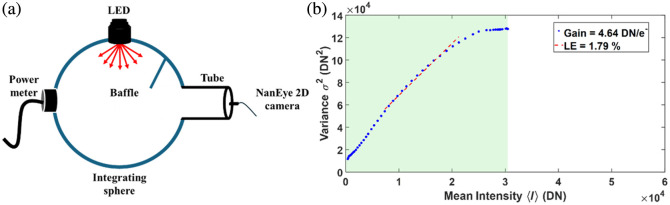
Experimental evaluation of NanEye2D camera conversion gain (g). (a) Experimental setup to obtain the PTC. (b) Estimation of the conversion gain and linearity error (LE) for the NanEye2D camera from the PTC. PTC was constructed from variance ($\sigma^{2}$) versus the mean intensity (<I>) in DN, with blue dots representing the measured data and the red dashed line indicating the linear fit. The slope of the linear fit yields the gain ($\mathrm{DN}/e^{-}$), and LE is calculated as the average deviation between the measured variance and the regression line.

This conversion gain was experimentally verified using a photon transfer curve (PTC) calibration approach.^47^ Figure 2(a) shows the experimental setup for evaluating the conversion gain of the NanEye2D camera. A calibrated integrating sphere with a 6-cm exit port (Labsphere, North Sutton, New Hampshire, United States) was used to generate spatially uniform illumination. A light-emitting diode (LED) at 730 nm (M730L5, Thorlabs, Newton, New Jersey, United States) was mounted to one input port, and a photodiode power meter (PM100D, Thorlabs, Newton, New Jersey, United States) was attached to the second port for monitoring light output. A baffle was placed inside the sphere to eliminate direct optical paths from the LED to the detector. The NanEye2D camera was placed 30 mm from the exit port using a custom three-dimensional printed matte black cylindrical tube, which minimized reflections and ensured >99% irradiance uniformity across the sensor.

Figure 2(b) shows the construction of the PTC based on a series of images acquired at increasing illumination levels. For each illumination level, a pixel-wise dark offset, calculated as the mean of 2500 dark frames recorded with the laser diodes off, was subtracted from every frame. The mean intensity (<I>) and corresponding variance ($\sigma^{2}$) were then computed in DN units across 100 frames using the entire detector array. A weighted linear regression was applied to the shot noise dominant region, defined by the conditions that the mean intensity remained below 70% of the sensor’s saturation level and the variance exceeded twice the estimated read noise. The slope of the fitted line yielded an experimentally measured camera gain of $4.64\;\mathrm{DN}/e^{-}$.

This experimental gain ($4.64\;\mathrm{DN}/e^{-}$) closely matched the theoretical conversion factor ($4.85\;\mathrm{DN}/e^{-}$), with 96% agreement. Accordingly, the experimentally derived gain of $4.64\;\mathrm{DN}/e^{-}$ was applied in subsequent DSCFO analyses to ensure accurate noise correction and speckle contrast calculation.

### 2.2 DSCFO Data Analysis

**Fig. 3:**
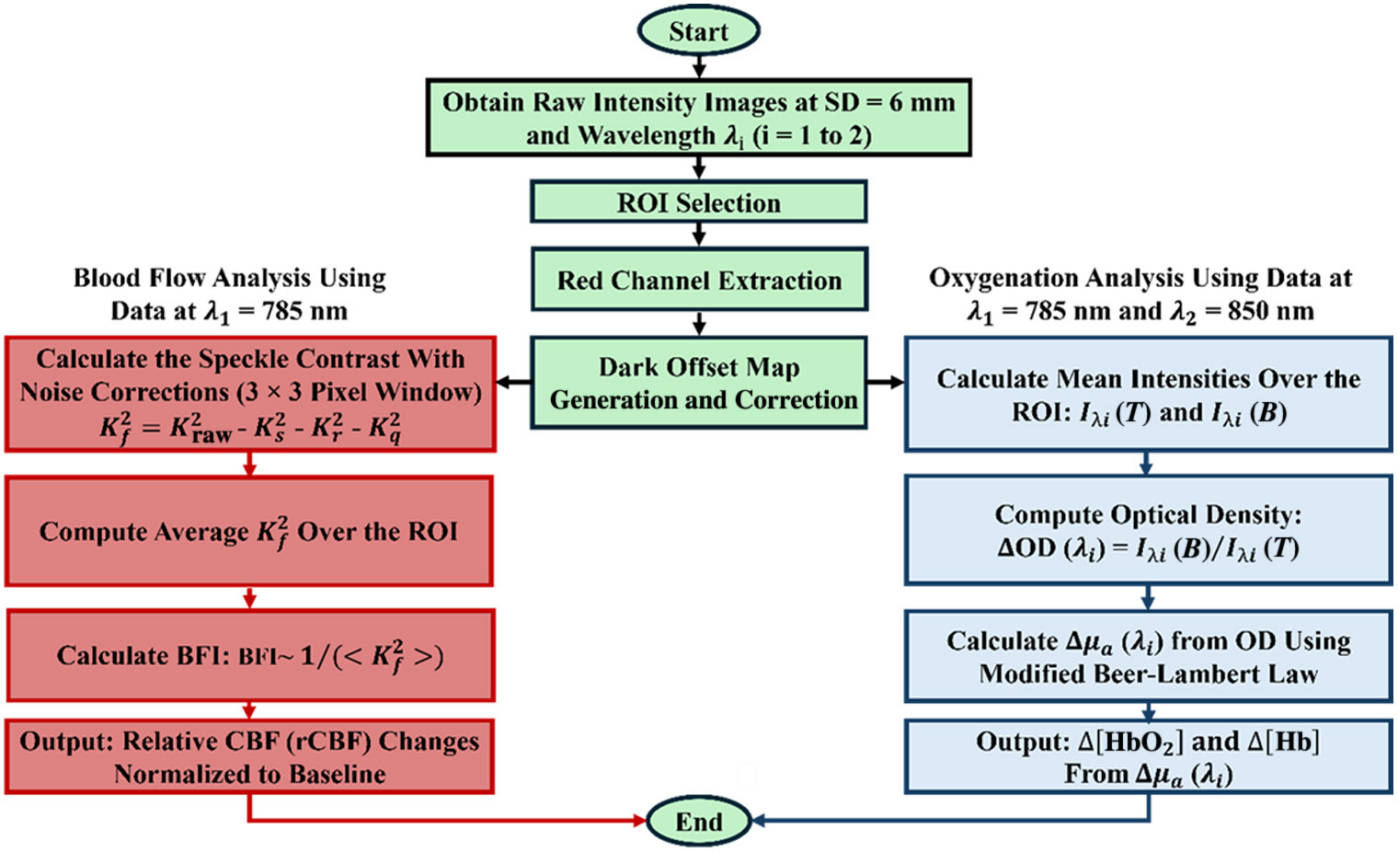
DSCFO data processing pipeline for quantifying changes in rCBF, $\Delta [{\mathrm{HbO}}_{2}]$, and Δ[Hb] from the recorded raw images. The red blocks detail the rCBF extraction using the wavelength at $\lambda_{1}=785\;\mathrm{nm}$. To obtain accurate BFI estimates from the measured speckle contrasts, noise corrections were implemented to address key sources of bias, including shot noise, read noise, and quantization noise. The blue blocks outline the calculation of $\Delta [{\mathrm{HbO}}_{2}]$ and Δ[Hb] using two wavelengths at $\lambda_{1}=785\;\mathrm{nm}$ and $\lambda_{2}=850\;\mathrm{nm}$.

Figure 3 shows a flowchart for data analysis to extract the blood flow index (BFI), relative CBF (rCBF), $\Delta [{\mathrm{HbO}}_{2}]$, and Δ[Hb]. BFI analysis was performed using the 785-nm light to maximize speckle SNR, as the NanEye camera exhibits higher spectral sensitivity near 785 nm than at 850 nm (83% at 780 nm versus 51% at 850 nm). Both the present results and our previous work show that blood flow measurements at the two wavelengths exhibit consistent dynamic trends, with only minor between-wavelength differences.^43^ Oxygenation analysis was performed using signals acquired at 785 and 850 nm. Laser powers at both wavelengths were adjusted to yield comparable detected intensity counts, compensating for differences in camera sensitivity and enabling accurate estimation of $\Delta [{\mathrm{HbO}}_{2}]$ and Δ[Hb].

#### 2.2.1 Cerebral blood flow

A fixed ROI of 60 × 60 pixels was selected at the center of each raw intensity image to encompass the cranial imaging area and exclude optical edge artifacts. The dark offset, determined as the average of 500 dark frames acquired with the laser diodes off ($I_{D}$), is subtracted from the raw intensity I to get the corrected intensity $I_{c}=I-I_{D}$. The fundamental speckle contrast ($K_{f}^{2}$) after shot, dark, and quantization noise corrections is given by Eq. (1):

$$K_{f}^{2}=K_{\mathrm{raw}}^{2}-K_{s}^{2}-K_{r}^{2}-K_{q}^{2}.$$ (1)

Here, the raw speckle contrast is defined as $K_{\mathrm{raw}}^{2}=\sigma^{2}/\langle I_{c}\rangle^{2}$, where σ and $\left\langle I_{c}\right\rangle$ are the standard deviation and mean of intensities within each 3×3 sliding window.^38^^,^^39^^,^^43^^,^^44^ The speckle contrast arising from photon shot noise is defined as $K_{s}^{2}=\;\sigma_{s}^{2}/\langle I_{c}\rangle^{2}$, where $\sigma_{s}^{2}=g\;I_{c}$ and g is the camera conversion gain. The contrast contribution from the camera’s read-out noise is defined as $K_{r}^{2}=\;\sigma_{r}^{2}/\langle I_{c}\rangle^{2}$, where $\sigma_{r}^{2}\;$ is the temporal variance of a series of 500 dark frames recorded in the absence of any illumination sources. The quantization noise bias is $K_{q}^{2}=\sigma_{q}^{2}\;/\langle I_{c}\rangle^{2}$, assuming the quantization-induced variance is $\sigma_{q}^{2}=\;1/12$.^48^ In calculating these speckle contrasts, a faster approach using MATLAB’s conv2 function was implemented^49^ instead of inefficient for-loops over each pixel window.

After subtracting all noise contributions from $K_{\mathrm{raw}}^{2}$ and calculating the noise-corrected $K_{f}^{2}$ using Eq. (1), the values were averaged over the ROI to yield $\left\langle K_{f}^{2}\right\rangle$. Although the precise relationship between the speckle contrast and the BFI is nonlinear, the BFI can be roughly estimated as the inverse of the averaged fundamental contrast squared in our analysis (BFI ∼ ($1/(\langle K_{f}^{2}\rangle \;$)).^50^ rCBF was then calculated by normalizing BFI to its baseline value prior to cerebral hemodynamic changes.

#### 2.2.2 Cerebral blood oxygenation

The $\Delta [{\mathrm{HbO}}_{2}]$ and Δ[Hb] were derived from the detected light intensities at two wavelengths ($\lambda_{1}=785\;\mathrm{nm}$ and $\lambda_{2}=850\;\mathrm{nm}$) using the modified Beer–Lambert law, as described in [Eqs. (25)].^51^ Raw intensity images were dark noise corrected, averaged over the ROI, and used to calculate relative changes in optical density ΔOD(λ)   and absorption coefficient $\Delta \mu_{a}(\lambda )$,

$$\Delta \mathrm{OD}(\lambda )=\;\ln(I_{\lambda B}/I_{\lambda T}),$$ (2)

$$\Delta \mu_{a}(\lambda )=\frac{\Delta \mathrm{OD}}{\rho {\mathrm{DPF}}_{\lambda }}.$$ (3)

Here, $I_{\lambda B}$ and $I_{\lambda T}$ were the corrected light intensities at baseline and at time T, respectively. The differential path factor (${\mathrm{DPF}}_{\lambda }$) accounted for the ratio of the mean photon path length to the source-detector separation (ρ), with values obtained from the literature.^52^^–^^54^ In this study, ${\mathrm{DPF}}_{785}=5$ and ${\mathrm{DPF}}_{850}=5$ were used based on the literature.^55^ The relative changes in [${\mathrm{HbO}}_{2}$] and [Hb] were calculated as follows:

$$\Delta [{\mathrm{HbO}}_{2}]=\;\frac{\varepsilon_{Hb}(\lambda_{1})\Delta \mu_{a}(\lambda_{2})-\varepsilon_{\mathrm{Hb}}(\lambda_{2})\Delta \mu_{a}(\lambda_{1})}{\varepsilon_{\mathrm{Hb}}(\lambda_{1})\varepsilon_{{\mathrm{HbO}}_{2}}(\lambda_{2})-\varepsilon_{{\mathrm{HbO}}_{2}}(\lambda_{1})\varepsilon_{\mathrm{Hb}}(\lambda_{2})},$$ (4)

$$\Delta [\mathrm{Hb}]=\;\frac{\varepsilon_{{\mathrm{HbO}}_{2}}(\lambda_{2})\Delta \mu_{a}(\lambda_{1})-\;\varepsilon_{{\mathrm{HbO}}_{2}}(\lambda_{1})\Delta \mu_{a}(\lambda_{2})}{\varepsilon_{\mathrm{Hb}}(\lambda_{1})\varepsilon_{{\mathrm{HbO}}_{2}}(\lambda_{2})\;-\;\varepsilon_{{\mathrm{HbO}}_{2}}(\lambda_{1})\varepsilon_{\mathrm{Hb}}(\lambda_{2})}.$$ (5)

Here, $\varepsilon_{\mathrm{Hb}}$ (λ) and $\varepsilon_{{\mathrm{HbO}}_{2}}$ (λ) are the extinction coefficients of Hb and ${\mathrm{HbO}}_{2}$, respectively.^56^

### 2.3 Animal Experimental Setup and Protocol

All animal procedures were conducted in accordance with the protocol approved by the Institutional Animal Care and Use Committee at the University of Kentucky. Two litters from time-pregnant rats were used in this study: rat #1 delivered nine pups, and rat #2 delivered eight pups. All neonatal rats (n=17) were randomly assigned to two groups: the sham group (n=7) and the IH group (n=10). The neonatal rats were chosen because they are cost-effective, easy to handle, and widely used in hypoxia-ischemia research. Furthermore, their brain development at early postnatal stages (e.g., postnatal day 8) parallels that of human late preterm infants, making them a relevant model for studying cerebral hemodynamic alterations under hypoxic and ischemic conditions.^57^

On postnatal day 8, rats were anesthetized with 1.25% isoflurane and secured in a stereotactic frame. Body temperature was maintained using a heating pad and intermittent infrared thermometer measurements to ensure proper thermal support [Fig. 1(d)]. The DSCFO probe, housed in a foam pad, was gently affixed to the scalp. Data were acquired at 1.6 Hz to monitor rCBF, $\Delta [{\mathrm{HbO}}_{2}]$, and Δ[Hb]. IH was induced by alternating the fractional inspired oxygen ($\%{\mathrm{FiO}}_{2}$) levels: 5-min normoxia (21% $O_{2}$), 10 cycles of 2-min hypoxic episode (8% $O_{2}$), and 2-min hyperoxic episode (100% $O_{2}$), then 14 min for recovery (∼60  min total). The hyperoxic phase was used as a standardized reoxygenation condition to generate reproducible hypoxia-hyperoxia transitions within a single session and to enable consistent comparison of cycle-dependent responses across animals. The 2-min hypoxia–hyperoxia cycle durations were selected to model repeated, controlled oxygen fluctuations in a minute-scale paradigm and to remain consistent with established neonatal IH models designed to capture clinically relevant features of apnea-associated oxygen instability.^58^^–^^60^ Because of the rapid transition among short-duration hypoxia–hyperoxia episodes, large accompanying fluctuations in tidal ${\mathrm{CO}}_{2}$ were not expected. The 10-cycle design provided repeated hypoxia-reoxygenation challenges within a single session to evaluate cycle-dependent response evolution while keeping total exposure feasible under stable anesthesia and physiological monitoring. Sham animals remained in normoxia with 10-min baseline monitoring.

Three animals were excluded due to motion artifacts (two IH and one sham) using visual inspection, video monitoring, and structural similarity index analysis (SSIM <0.97). Final analysis included eight IH and six sham rats.

### 2.4 Statistical Analysis

The repeated measures analysis of variance (ANOVA) was conducted to evaluate the effects of hypoxia–hyperoxia cycles on rCBF, $\Delta [{\mathrm{HbO}}_{2}]$, and Δ[Hb] in neonatal rats. This analysis enabled examination of within-subject effects across different time points, including baseline, IH cycles, and recovery. Mauchly’s test of sphericity was conducted to assess the assumption of sphericity. When the test was not significant, the sphericity assumption was considered met, and the sphericity-assumed overall p-value was reported. Otherwise, the Greenhouse–Geisser correction was applied. Further, if the overall p-value is significant, *post hoc* pairwise comparisons with the least significant difference tests were performed to identify specific differences between two time points. All statistical analyses were performed using IBM SPSS Statistics, and results were considered statistically significant at p<0.05.

## 3 Results

### 3.1 Individual Cerebral Hemodynamic Responses to Sham and IH

**Fig. 4:**
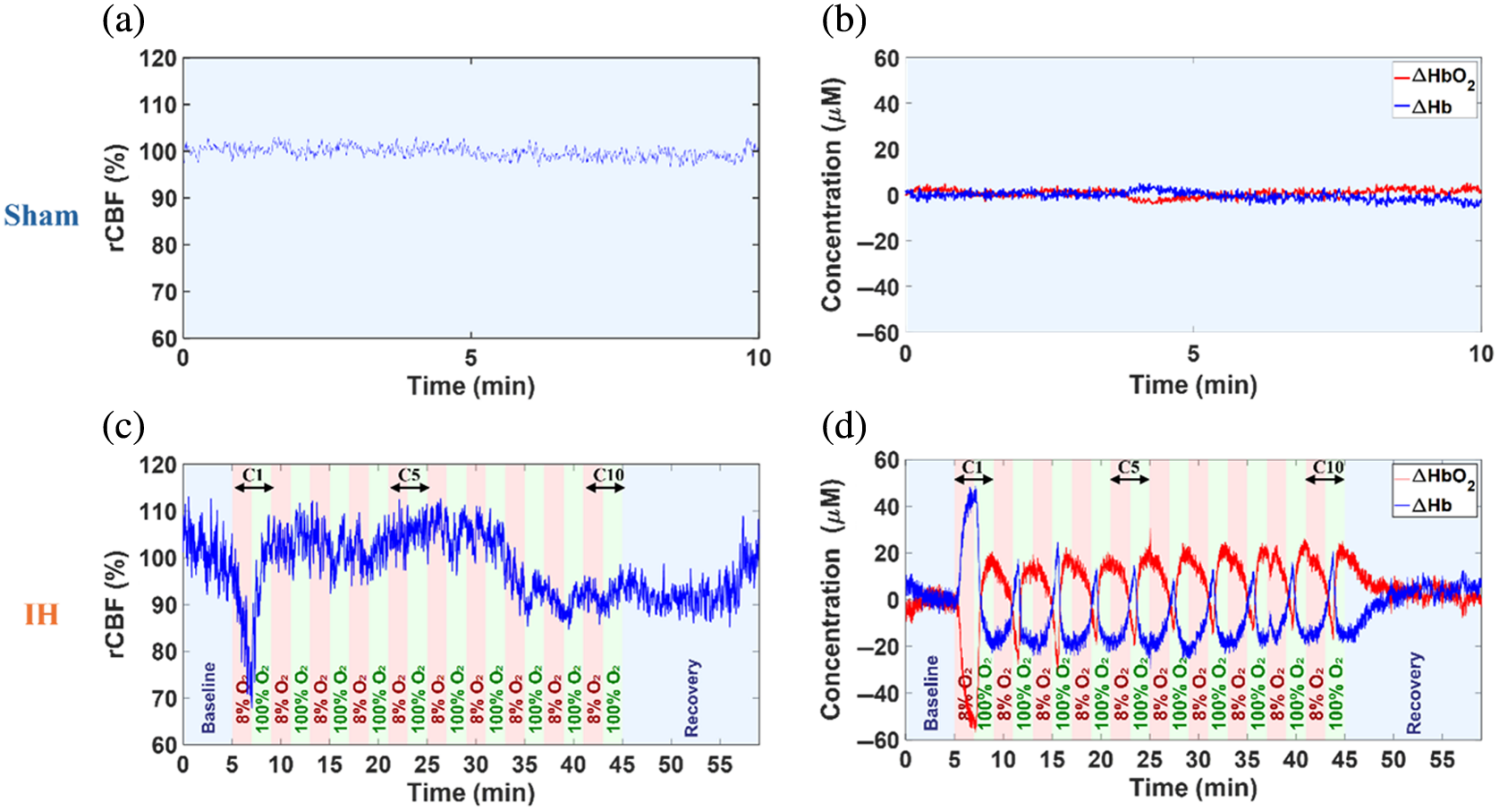
Individual time-course cerebral hemodynamic changes continuously measured by DSCFO. (a) and (b) rCBF, $\Delta [{\mathrm{HbO}}_{2}]$, and Δ[Hb] remained stable during 10-min monitoring in a representative sham rat. (c) rCBF decreased during hypoxia and increased during hyperoxia in a representative IH rat. (d) $\Delta [{\mathrm{HbO}}_{2}]$ decreased and Δ[Hb] increased during hypoxia, reversing during hyperoxia in the representative IH rat.

Figure 4 shows representative time-course variations in rCBF, $\Delta [{\mathrm{HbO}}_{2}]$, and Δ[Hb] for sham and IH groups. Minimal fluctuations in rCBF, $\Delta [{\mathrm{HbO}}_{2}]$, and Δ[Hb] were observed during 10 min of DSCFO monitoring in a sham rat under normoxic isoflurane anesthesia [Figs. 4(a) and 4(b)], indicating stable cerebral perfusion and oxygenation in the absence of hypoxia.

In contrast, an illustrative IH rat undergoing 10 hypoxia-hyperoxia cycles (C1 to C10) showed marked periodic fluctuations in rCBF, $\Delta [{\mathrm{HbO}}_{2}]$, and Δ[Hb] [Figs. 4(c) and 4(d)]. During C1, when normoxia was abruptly switched to hypoxic 8% $O_{2}$ (in $N_{2}$), rCBF and $\Delta [{\mathrm{HbO}}_{2}]$ exhibited the steepest declines while Δ[Hb] rose sharply, reflecting cerebral hypoxic stress from reduced oxygen delivery. The subsequent hyperoxia reversed these changes. Across C2 to C10, the same pattern persisted but with a smaller amplitude than at C1. In each hypoxic episode, rCBF and $\Delta [{\mathrm{HbO}}_{2}]$ decreased while Δ[Hb] increased, with paired hyperoxia producing opposite rebounds. Following C10, cerebral hemodynamic variables gradually returned toward baseline, and by the last 2 min of recovery, all measures were nearly restored.

### 3.2 Group Cerebral Hemodynamic Responses to Sham and IH

**Fig. 5:**
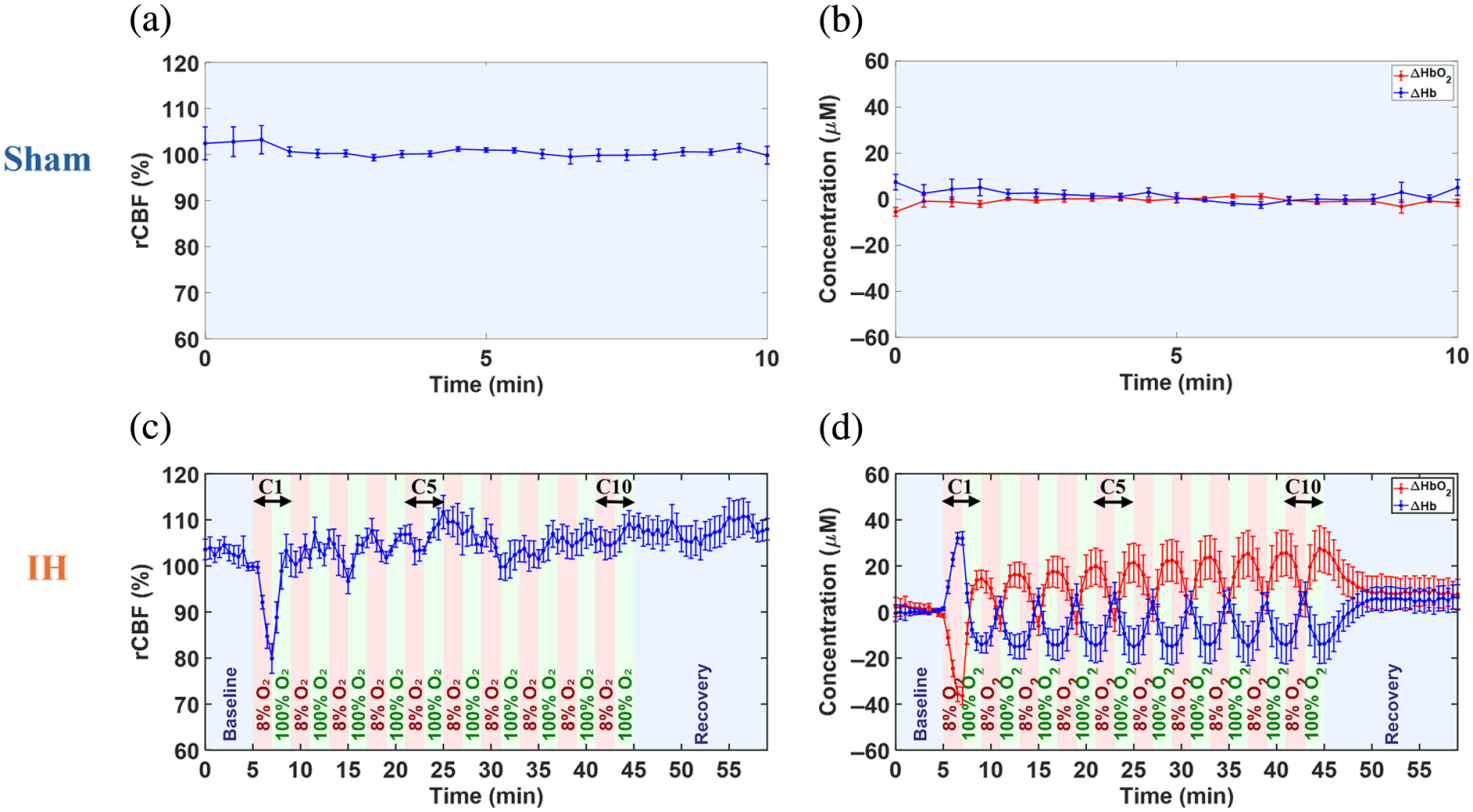
Group-averaged cerebral hemodynamic changes in six sham and eight IH rats. Data were normalized to their initial baseline values and presented as mean ± standard errors (error bars). (a) and (b) Sham rats (n=6) show stable $\Delta [{\mathrm{HbO}}_{2}]$, Δ[Hb], and rCBF throughout the monitoring period of 10 min. (c) IH rats (n=8) exhibited significant fluctuations in rCBF across 10 IH cycles (C1 to C10), showing a decline during hypoxia followed by an increase during hyperoxia. (d) IH rats showed a significant decrease in $\Delta [{\mathrm{HbO}}_{2}]$ during hypoxia and an increase during hyperoxia, whereas Δ[Hb] displayed an inverse pattern.

Figure 5 presents group-averaged cerebral hemodynamic fluctuations across all experimental rats, including eight from the IH group and six from the sham group. Time-course results are presented as the mean values ± standard errors (error bars). Every 48 consecutive data points (∼30  s at 1.6 Hz) were averaged and plotted with corresponding error bars to improve readability.

Sham rats demonstrated stable cerebral hemodynamics under normoxia, with rCBF=100.63±0.07%, $\Delta [{\mathrm{HbO}}_{2}]=-0.57\pm 0.59\;\mathrm{\mu M}$, and Δ[Hb]=1.51±0.77  μM [Figs. 5(a) and 5(b)]. In contrast, IH rats exhibited pronounced periodic fluctuations across the C1-C10 hypoxia-hyperoxia cycles [Figs. 5(c) and 5(d)]. During C1, hypoxia (8% $O_{2}$ in $N_{2}$) induced the largest changes: rCBF dropped to −79.90±3.23%, $\Delta [{\mathrm{HbO}}_{2}]$ decreased to −36.39±3.75  μM, and Δ[Hb] increased to 31.98±2.89  μM. The subsequent hyperoxia reversed these effects, with rCBF rebounding to 101.21±3.49%, $\Delta [{\mathrm{HbO}}_{2}]$ rising to 14.38±3.79  μM, and Δ[Hb] falling to −14.11±3.64  μM.

Across C2 to C10, the same hypoxia–hyperoxia pattern repeated. rCBF fluctuations were less rhythmic than those of $\Delta [{\mathrm{HbO}}_{2}]$ and Δ[Hb]. Hypoxia generally reduced rCBF and $\Delta [{\mathrm{HbO}}_{2}]$ while increasing Δ[Hb], with hyperoxia producing the opposite effects. Cycle-to-cycle variability was modest compared with that at C1. At C5, hypoxia decreased rCBF by −3.40±2.07% and $\Delta [{\mathrm{HbO}}_{2}]$ by −23.27±9.31  μM while increasing Δ[Hb] by 22.57±8.94  μM relative to the preceding C4 hyperoxia endpoint. The subsequent hyperoxia elevated rCBF by 8.26±3.69% and $\Delta [{\mathrm{HbO}}_{2}]$ by 17.95±9.74  μM while reducing Δ[Hb] by −23.14±9.02  μM. At C10, hypoxia decreased rCBF by −0.43±3.85% and $\Delta [{\mathrm{HbO}}_{2}]$ by −22.32±11.20  μM while increasing Δ[Hb] by 22.45±9.61  μM relative to the C9 hyperoxia endpoint. The following hyperoxia raised rCBF by 3.09±3.92% and $\Delta [{\mathrm{HbO}}_{2}]$ by 23.45±11.18  μM while reducing Δ[Hb] by −22.04±9.72  μM. In the final 2 min of the recovery, group results approached their baselines with mild overshoots (rCBF=107.96±2.32%, $\Delta [{\mathrm{HbO}}_{2}]=7.76\pm 6.43\;\mathrm{\mu M}$, Δ[Hb]=6.38±5.44  μM).

### 3.3 Statistical Analysis Results of Group Cerebral Hemodynamic Responses to Sham and IH

**Fig. 6:**
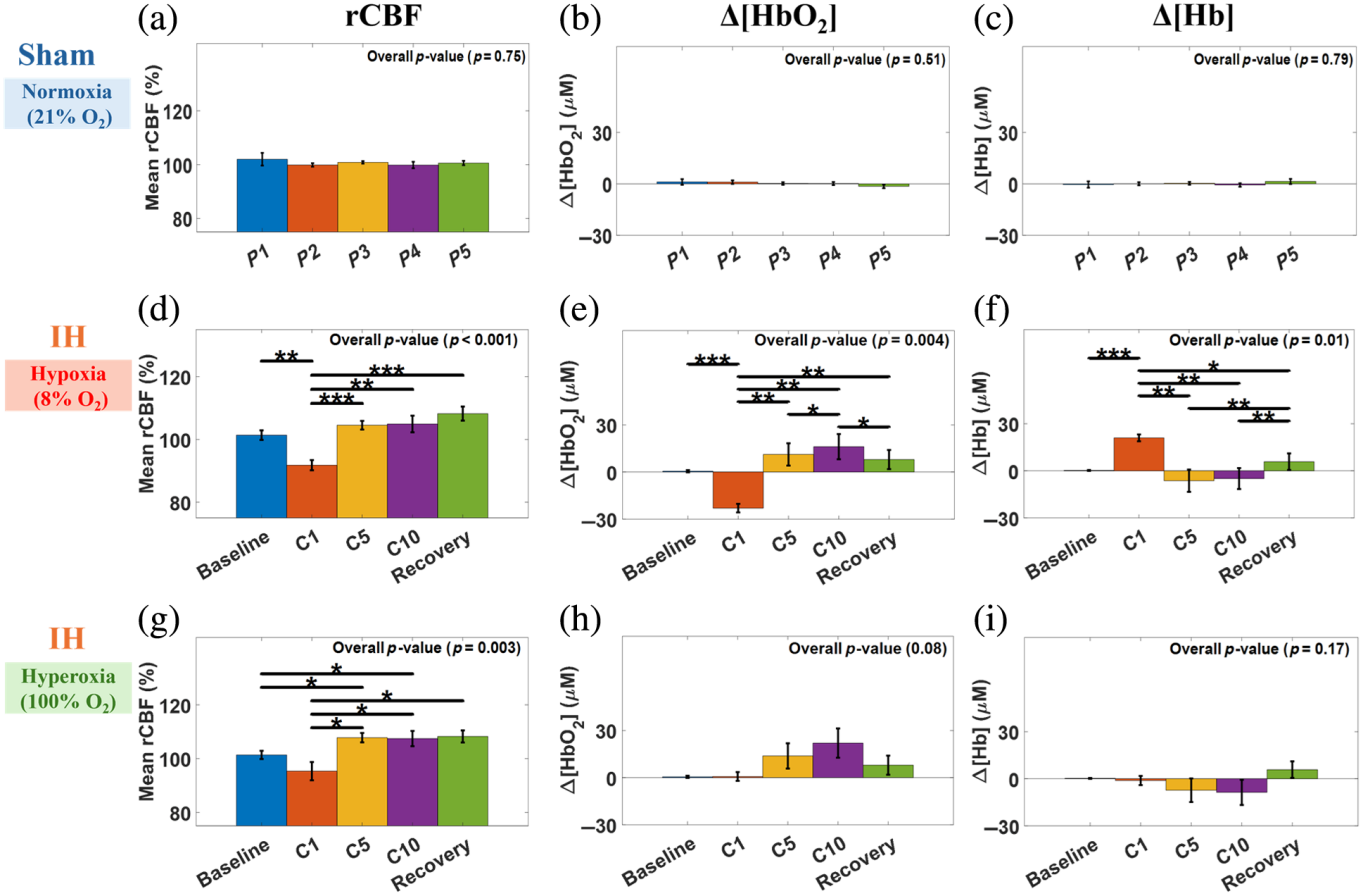
Average within-episode changes in rCBF, $\Delta [{\mathrm{HbO}}_{2}]$, and Δ[Hb] in the sham and IH groups. Data were normalized to their initial baseline values, averaged over the reporting period, and presented as mean ± standard errors (error bars). Positive values ($\Delta [{\mathrm{HbO}}_{2}]$ and Δ[Hb]) or values above 100% (rCBF) denote increases above baseline, and negative values ($\Delta [{\mathrm{HbO}}_{2}]$ and Δ[Hb]) or values below 100% (rCBF) denote decreases below baseline. (a)–(c) Sham rats (n=6) showed stable rCBF, $\Delta [{\mathrm{HbO}}_{2}]$, and Δ[Hb] across five monitoring periods (P1–P5) during 10 min of normoxia. (d)–(f) IH rats (n=8) exhibited significant changes in rCBF, $\Delta [{\mathrm{HbO}}_{2}]$, and Δ[Hb] during hypoxia (8% $O_{2}$) across five reporting periods: baseline, C1, C5, C10, and recovery. (g)–(i) IH rats exhibited significant changes in rCBF, $\Delta [{\mathrm{HbO}}_{2}]$, and Δ[Hb] during hyperxia (100% $O_{2}$) across five reporting periods. *p<0.05, **p<0.01, and ***p<0.001.

Figure 6 shows the time-course changes in rCBF, $\Delta [{\mathrm{HbO}}_{2}]$, and Δ[Hb] relative to their initial baselines in both the sham and IH groups. In the sham group (n=6), five consecutive 2-min reporting periods (P1 to P5) within the 10-min normoxic condition were pre-specified to facilitate meaningful time-course comparisons and minimize the number of multiple statistical tests [Figs. 6(a)–6(c)]. Repeated measures ANOVA across these periods revealed no significant changes in rCBF (p=0.75), $\Delta [{\mathrm{HbO}}_{2}]$ (p=0.51), or Δ[Hb](p=0.79).

**Fig. 7:**
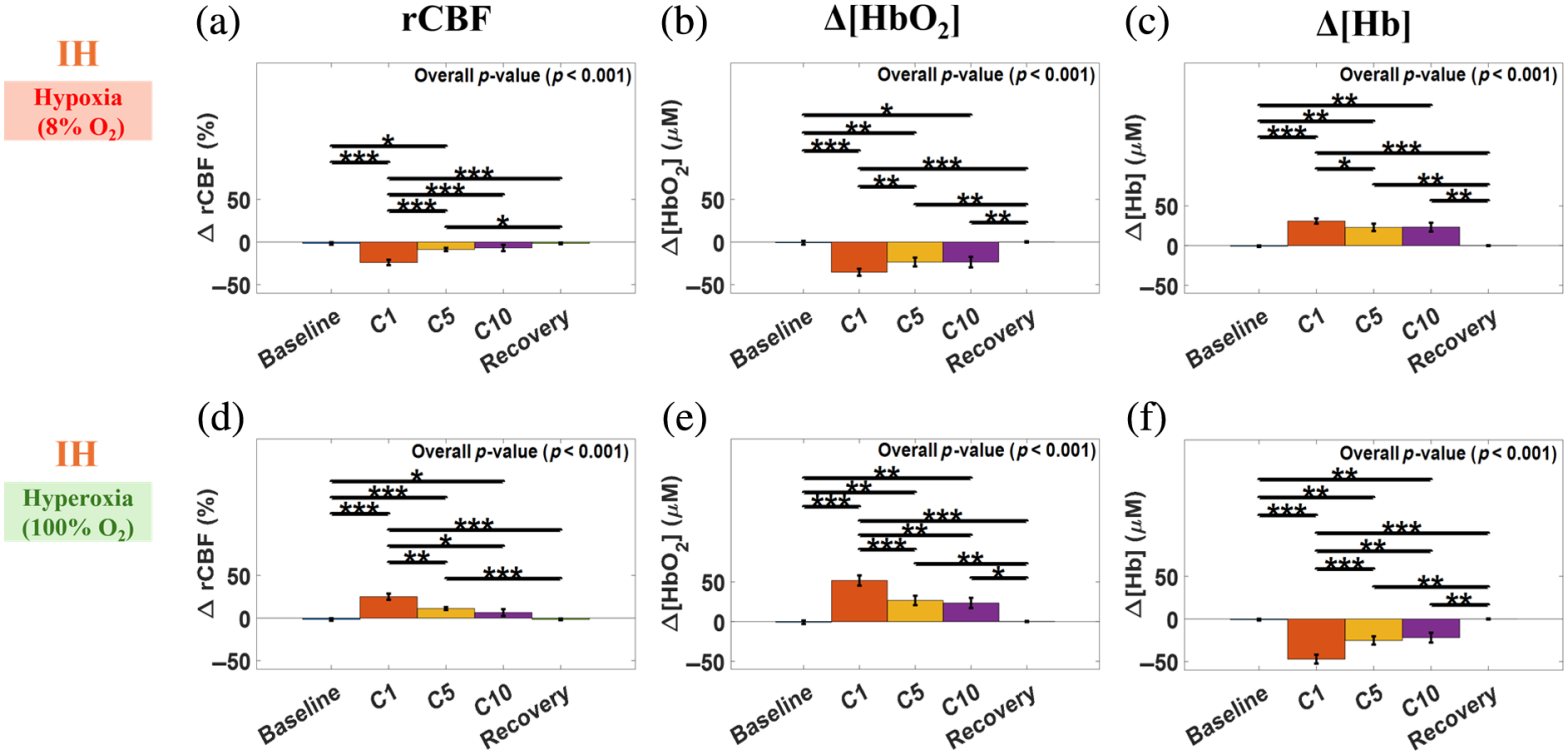
Maximum within-episode changes in rCBF, $\Delta [{\mathrm{HbO}}_{2}]$, and Δ[Hb] in the IH group (n=8). The maximum change during each IH episode (hypoxia or hyperoxia) was calculated as the difference between the maximum and minimum values within that episode, defined as positive for increases and negative for decreases. Data were presented as mean ± standard errors (error bars). (a)–(c) IH rats exhibited significant decreases in rCBF and $\Delta [{\mathrm{HbO}}_{2}]$ and significant increases in Δ[Hb] during hypoxia (8% $O_{2}$) across five reporting periods: baseline, C1, C5, C10, and recovery. (d)–(f) IH rats exhibited significant increases in rCBF and $\Delta [{\mathrm{HbO}}_{2}]$ and significant decreases in Δ[Hb] during hyperoxia (100% $O_{2}$) across the same reporting periods. *p<0.05, **p<0.01, and ***p<0.001.

In the IH group (n=8), five consecutive reporting periods (baseline, C1, C5, C10, and recovery) were pre-specified to facilitate meaningful time-course comparisons. Baseline served as the normalization point (rCBF = 100%; $\Delta [{\mathrm{HbO}}_{2}]$ and Δ[Hb]=0  μM), C1 captured the acute response, C5 indexed mid-protocol adaptation, C10 reflected late cumulative effect, and recovery assessed post-challenge rebound. Data were analyzed with two complementary approaches to quantify distinct cerebral response features to the IH. The episode-averaged approach assessed average cerebral hemodynamic responses relative to baseline within the hypoxic or hyperoxic episode (Fig. 6). Positive values ($\Delta [{\mathrm{HbO}}_{2}]$ and Δ[Hb]) or values above 100% (rCBF) denote increases above baseline, whereas negative values ($\Delta [{\mathrm{HbO}}_{2}]$ and Δ[Hb]) or values below 100% (rCBF) denote decreases below baseline. The episode-maximum-change approach quantifies the difference between the maximum and minimum values within the hypoxic or hyperoxic episode, defined as positive for increases and negative for decreases (Fig. 7). Using the episode-averaged approach, repeated-measures ANOVA tests during hypoxic episodes [Figs. 6(d)–6(f)] revealed significant differences in rCBF, $\Delta [{\mathrm{HbO}}_{2}]$, and Δ[Hb] across the five reporting periods, with overall *p*-values of <0.001, 0.004, and 0.01, respectively. rCBF and $\Delta [{\mathrm{HbO}}_{2}]$ at C1 were significantly lower than their baselines, whereas Δ[Hb] was significantly higher. rCBF and $\Delta [{\mathrm{HbO}}_{2}]$ at C5, C10, and during recovery were significantly higher than at C1, whereas Δ[Hb] at C5, C10, and during recovery were significantly lower than at C1. $\Delta [{\mathrm{HbO}}_{2}]$ at C10 was significantly higher than at C5. $\Delta [{\mathrm{HbO}}_{2}]$ at recovery was significantly lower than at C10. Δ[Hb] at recovery was significantly higher than at C5 and C10.

During hyperoxic episodes [Figs. 6(g)–6(i)], repeated-measures ANOVA tests revealed significant differences across reporting periods for rCBF (p=0.003) but not for $\Delta [{\mathrm{HbO}}_{2}]$ (p=0.08) and Δ[Hb] (p=0.17). rCBF at C5 and C10 was significantly higher than its baseline. rCBF at C5 and C10, and during recovery, was significantly higher than at C1.

Using the episode-maximum-change approach, repeated-measures ANOVA tests during hypoxic episodes [Figs. 7(a)–7(c)] detected significant differences across reporting periods for rCBF, $\Delta [{\mathrm{HbO}}_{2}]$, and Δ[Hb], with overall *p*-values of <0.001 for all parameters. Hypoxia consistently decreased rCBF and $\Delta [{\mathrm{HbO}}_{2}]$ and increased Δ[Hb], but their amplitudes declined from C1 to C10, reflecting attenuation of instantaneous cerebral fluctuations across cycles.

During hyperoxic episodes [Figs. 7(d)–7(f)], repeated-measures ANOVA tests detected significant differences across reporting periods for rCBF, $\Delta [{\mathrm{HbO}}_{2}]$, and Δ[Hb], with overall *p*-values of <0.001 for all parameters. Hyperoxia consistently increased rCBF and $\Delta [{\mathrm{HbO}}_{2}]$ and decreased Δ[Hb], but their amplitudes diminished from C1 to C10, suggesting attenuation of instantaneous cerebral fluctuations across cycles. In addition, the amplitude increases of rCBF and $\Delta [{\mathrm{HbO}}_{2}]$ at C1 were significantly larger than at C5 and C10, and the amplitude decrease of Δ[Hb] at C1 was significantly larger than at C5 and C10.

## 4 Discussion and Conclusions

Intermittent hypoxia is common in preterm infants and contributes to brain injury, creating a need for continuous monitoring of CBF and cerebral oxygenation at the bedside.^61^^,^^62^ Neonatal rodent models approximate premature brain development, allowing controlled IH paradigms and rigorous validation, making them well-suited for testing cerebral monitoring tools and potential interventions. Previous neonatal rodent studies primarily assessed IH- or prolonged-hypoxia-induced brain injury using histology and systemic physiological measures (e.g., heart rate, respiration, oxygen saturation, and blood pressure).^58^^–^^60^

One related study quantified CBF changes in neonatal rats during prolonged hypoxia (60 min) using laser Doppler flowmetry (LDF), without concurrent cerebral oxygenation measurements.^63^ In that study, 7-day-old rat pups were exposed to 60 min of hypoxia (8% O_2_) followed by 60 min of reoxygenation (21% $O_{2}$), and only phase-averaged responses were reported, precluding resolution of rapid post-transition dynamics. rCBF decreased to 93±3% of baseline during hypoxia and increased to 124±3% during reoxygenation.^63^ In contrast, our protocol employed repeated 2-min cycles of hypoxia (8% $O_{2}$) and hyperoxia (100% $O_{2}$), enabling resolution of rapid within-episode rCBF dynamics. Because of these fundamental differences in temporal design, rCBF magnitudes are not directly comparable; nevertheless, the consistent direction of responses across studies supports the physiological validity of the DSCFO-derived rCBF trends. Moreover, LDF is limited to superficial microvasculature, samples a small cortical area, and requires invasive skull preparation.

Continuous monitoring of both CBF and cerebral oxygenation during IH is critical, as oxygenation alone cannot differentiate reduced CBF from increased cerebral oxygen utilization. Combining the two provides mechanistic insight, enhances event detection, and quantifies cerebrovascular reactivity. To our knowledge, no study in neonatal rodents has simultaneously measured both quantities during IH. To address this gap, we developed a wearable DSCFO system and validated it in human forearms as well as in the brains of large neonatal piglets and human neonates.^43^^,^^44^ For this study, the DSCFO system was adapted for use in small neonatal rats. Two compact laser diodes and a tiny CMOS camera were integrated into a miniaturized fiber-free probe for continuous cerebral monitoring during IH (Fig. 1). The DSCFO device was optimized and characterized for performance and footprint (Fig. 2). A dedicated processing pipeline was implemented to extract rCBF, $\Delta [{\mathrm{HbO}}_{2}]$, and Δ[Hb] from raw speckle data obtained by the DSCFO (Fig. 3). The optimized system was evaluated in neonatal rats during IH to monitor dynamic changes in rCBF, $\Delta [{\mathrm{HbO}}_{2}]$, and Δ[Hb], demonstrating its sensitivity to hypoxia–hyperoxia induced cerebral hemodynamic variations.

As expected, sham rats maintained stable cerebral hemodynamics under normoxia, whereas IH rats exhibited pronounced periodic fluctuations in cerebral hemodynamics across the C1 to C10 hypoxia–hyperoxia cycles (Figs. 4 and 5). During C1, transition to hypoxia (8% $O_{2}$) in IH rats induced the largest changes in rCBF, $\Delta [{\mathrm{HbO}}_{2}]$, and Δ[Hb], which were reversed by subsequent hyperoxia (100% $O_{2}$). Across C2 to C10, similar episode-dependent hypoxia–hyperoxia patterns recurred, though rCBF fluctuations were less rhythmic than those of $\Delta [{\mathrm{HbO}}_{2}]$ and Δ[Hb], and cycle-to-cycle variability was modest compared with C1. The pronounced initial cerebral hemodynamic response and subsequent attenuation likely reflect physiological adaptation to repeated IH exposure. In the last 2 min of recovery, group-averaged signals returned toward baseline levels with mild overshoots.

The episode-averaged analysis revealed that the initial hypoxic episode (C1) caused significant cerebral deoxygenation from baseline (Figs. 6(d)–6(f)], which was reversed by the subsequent hyperoxic episode [Figs. 6(g)–6(i)]. Particularly, repeated hyperoxic episodes across C2 to C10 circles resulted in restored cerebral hemodynamics, characterized by significantly higher rCBF at C5 and $\Delta [{\mathrm{HbO}}_{2}]$ at C10, compared with baseline. These findings suggest that timely and efficient hyperoxia (100% $O_{2}$) can reverse the hypoxic stress, thus protecting the brain.

The episode-maximum-change analysis showed that hypoxic episodes induced significant instantaneous decreases in rCBF and Δ[HbO_2_] and increases in Δ[Hb] [Figs. 7(a)–7(c)], whereas hyperoxic episodes produced significant but reversed changes [Figs. 7(d)–7(f)]. For all three parameters, response amplitudes were greatest at C1 and progressively declined through subsequent cycles. This attenuation across C2 to C10 was likely due to residual oxygen carried over from previous hyperoxic episodes, replenishing the circulatory oxygen buffer.^64^^,^^65^ Repeated exposure to 100% $O_{2}$ during hyperoxia increased cerebral oxygen buffering and venous saturation, thereby attenuating subsequent deoxygenation and rCBF responses. Because NICU patients receive titrated oxygen concentrations ranging from 40% to 100% to maintain adequate ${\mathrm{SpO}}_{2}$,^66^^,^^67^ 100% $O_{2}$ was used in the present study as an upper-bound reoxygenation condition to ensure consistent comparisons across cycles. Nonetheless, these pronounced cerebral hemodynamic fluctuations during IH may contribute to brain injury, such as hemorrhage from large vessel rupture or micro-bleeding at the microvascular level, although histological confirmation is required.^68^^–^^70^

The episode-averaged and episode-maximum-change metrics provide complementary views of cerebral responses during IH. The episode-averaged results (Fig. 6) quantify mean cerebral hemodynamic levels within each 2-min episode and are more indicative of cumulative metabolic stress (when below baseline) or recovery (when above baseline). In contrast, the episode-maximum-change results (Fig. 7) capture the peak magnitude of transient cerebral hemodynamic fluctuations within each episode, which may adversely impact the brain regardless of whether they occur above or below baseline. Together, the two complementary analyses revealed episode-dependent protective and disruptive mechanisms, offering insight to guide future interventions based on continuous cerebral hemodynamic monitoring.

Our next phase will extend this work into a systematic developmental framework. To accommodate head growth in longitudinal studies, we will incorporate an adjustable head stage and interchangeable probes with different S-D separations, as implemented previously.^41^ We also plan to translate the technique to larger animal models, such as neonatal piglets, where measurements can be performed without anesthesia, enabling continuous monitoring and facilitating eventual clinical translation to preterm infants, including IH studies.

Future experiments will increase the number and duration of IH episodes to evaluate their cumulative impact on the brain. We will quantify response kinetics, such as time to peak change and recovery half-time during reoxygenation, using extended reoxygenation windows to avoid truncation bias. These kinetic metrics will provide insight into reoxygenation efficiency and oxygen delivery stress across protocols. Finally, DSCFO measurements will be correlated with neurological and histological outcomes to validate brain injury and inform intervention strategies through continuous cerebral hemodynamic assessment in neonatal animals and, ultimately, human infants.

In conclusion, this study demonstrates an affordable, noninvasive, wearable, fiber-free DSCFO system for continuous and simultaneous monitoring of rCBF, $\Delta [{\mathrm{HbO}}_{2}]$, and Δ[Hb] during IH in neonatal rats. The system effectively captured hypoxia-induced cerebral deoxygenation and hyperoxia-driven cerebral oxygen restoration, confirming its technical feasibility and biological sensitivity for bedside cerebral monitoring.

## Data Availability

All codes and data supporting the findings of this study are available from the corresponding author upon reasonable request.
